# Physical activity and dietary behaviour in a population-based sample of British 10-year old children: the SPEEDY study (Sport, Physical activity and Eating behaviour: Environmental Determinants in Young people)

**DOI:** 10.1186/1471-2458-8-388

**Published:** 2008-11-14

**Authors:** Esther MF van Sluijs, Paula ML Skidmore, Kim Mwanza, Andrew P Jones, Alison M Callaghan, Ulf Ekelund, Flo Harrison, Ian Harvey, Jenna Panter, Nicolas J Wareham, Aedin Cassidy, Simon J Griffin

**Affiliations:** 1 Medical Research Council Epidemiology Unit, Institute of Metabolic Science, Box 285, Addenbrookes Hospital, Hills Road, Cambridge, CB2 0QQ, UK; 2 School of Medicine, Health Policy and Practice, University of East Anglia, Norwich, NR4 7JT, UK; 3 School of Environmental Sciences, University of East Anglia, Norwich, NR4 7JT, UK

## Abstract

**Background:**

The SPEEDY study was set up to quantify levels of physical activity (PA) and dietary habits and the association with potential correlates in 9–10 year old British school children. We present here the analyses of the PA, dietary and anthropometry data.

**Methods:**

In a cross-sectional study of 2064 children (926 boys, 1138 girls) in Norfolk, England, we collected anthropometry data at school using standardised procedures. Body mass index (BMI) was used to define obesity status. PA was assessed with the Actigraph accelerometer over 7 days. A cut-off of ≥ 2000 activity counts was used to define minutes of moderate-to-vigorous PA (MVPA). Dietary habits were assessed using the Health Behaviour in School Children food questionnaire. Weight status was defined using published international cut-offs (Cole, 2000). Differences between groups were assessed using independent t-tests for continuous data and chi-squared tests for categorical data.

**Results:**

Valid PA data (>500 minutes per day on ≥ 3 days) was available for 1888 children. Mean (± SD) activity counts per minute among boys and girls were 716.5 ± 220.2 and 635.6 ± 210.6, respectively (p < 0.001). Boys spent an average of 84.1 ± 25.9 minutes in MVPA per day compared to 66.1 ± 20.8 among girls (p < 0.001), with an average of 69.1% of children accumulating 60 minutes each day. The proportion of children classified as overweight and obese was 15.0% and 4.1% for boys and 19.3% and 6.6% for girls, respectively (p = 0.001). Daily consumption of at least one portion of fruit and of vegetables was 56.8% and 49.9% respectively, with higher daily consumption in girls than boys and in children from higher socioeconomic backgrounds.

**Conclusion:**

Results indicate that almost 70% of children meet national PA guidelines, indicating that a prevention of decline, rather than increasing physical activity levels, might be an appropriate intervention target. Promotion of daily fruit and vegetable intake in this age group is also warranted, possibly focussing on children from lower socioeconomic backgrounds.

## Background

A lack of physical activity and poor dietary habits are believed to be the major contributors to the current rise in childhood obesity [1-3]. Both behaviours have also been shown to have independent associations with other health problems during childhood, including metabolic impairments [4-8] and poor skeletal health [9,10]. They also tend to co-exist both in adults and children [11,12]. Increasing physical activity and improving dietary habits in childhood have therefore been identified as targets for future public health policy [1,13]. To be able to effectively promote changes in these complex health behaviours, reliable and valid data are needed about current patterns, and the demographic and modifiable factors that are most strongly associated with them. This will aid the identification of populations at risk and the development of interventions to promote change in these health behaviours.

To date, the extent of the problem of physical inactivity and unhealthy dietary habits in children is largely unknown. Descriptive data on patterns and levels of physical activity assessed using valid and reliable measures in large population-based samples is scarce, especially in children [14]. Previous studies have reported mixed results, with the proportion of children achieving the recommended guideline of at least one hour of moderate-to-vigorous intensity physical activity (MVPA) each day [13] varying from 2.5 to 97% [13,15-18]. This large variation mainly depends on the population studied and the variation in data processing methods applied. However, most studies indicate that physical activity levels decline with age, especially during late primary school years and throughout secondary school [19-21], making this a potentially important period for health promotion interventions. Although there is some information available on the nutritional content of the diet of primary school-aged children (e.g. [22,23]), little is known about their dietary behaviour such as food choice. Previous studies either primarily focussed on secondary school-aged children [24] or were conducted a decade ago [25]. In order to develop behavioural interventions targeted at improving dietary habits and potentially prevent obesity in primary school-aged children, up-to-date information about the dietary habits is required.

The SPEEDY study (Sport, Physical activity and Eating behaviour: Environmental Determinants in Young people) was established to examine physical activity levels and dietary behaviour in a large population-based sample of British 9–10 year old children, and to investigate the individual and collective factors associated with these behaviours. The first aim of the SPEEDY study is to assess the extent of the problem of physical inactivity and unhealthy dietary habits. With its population-based sampling strategy, large sample size and detailed assessment of both physical activity and diet, the SPEEDY study will add to the existing evidence base. The aim of the current paper is to describe the SPEEDY study and the levels of physical activity and dietary habits of its participants.

## Methods

### Study sample

#### School recruitment

Schools in the county of Norfolk, South-East England, were sampled purposively to achieve heterogeneity in location. For logistical purposes, only schools with at least 12 Year 5 pupils (aged 9–10 years) were sampled. Head teachers at selected schools were first sent an invitation letter and an information leaflet, and then contacted to arrange a face-to-face visit. In this 10-minute visit, details were provided about the aims of the study, the invitation and measurement procedures, the degree of school involvement required, and potential benefits of participation. An information pack was provided at the visit, containing a copy of the ethical approval, examples of all study materials, and information leaflets for all Year 5 teachers. If they agreed that their school could take part, head teachers were asked to send the study team a signed acceptance letter. Upon request, a smaller sister school of one of the participating schools (a smaller school run under shared administration) was allowed to participate. School recruitment was completed prior to the start of data collection in April 2007.

#### Participant recruitment

Ethical approval for the SPEEDY study was obtained from the University of East Anglia local research ethics committee. Teams of two research assistants visited participating schools two weeks before the measurement date and introduced the SPEEDY study to all Year 5 children. Children were given an information pack containing a leaflet for themselves, a letter for their parents/guardians, and a consent form. Children were instructed to return the completed consent form to the school if they were willing to participate. A classroom poster was left at the school to remind the children of when the data collection was taking place. Three days before the measurement visit, a member of the research team contacted the school to assess response rates and to ask the class teacher to reinforce the message that children would not be able to participate without completed consent. Only children with a fully completed consent form (signed by both a parent/guardian and the child) on the day of measurement were included in the study. An average of 168 children (range: 121 to 242 per week) was recruited into the study each week with an aim of recruiting 2000 children over a 12-week period.

### Measurement procedures

Data collection was performed during the Summer term of 2007 (April to July). Teams of two or more trained research assistants conducted measurements at participating schools according to standard operating procedures. They visited between five and 10 schools each week. All participating children self-completed their questionnaires with at least one research assistant available for assistance. Completeness of the questionnaire was determined on-site for quality control. Concurrently, research assistants measured anthropometry, obtained a saliva sample (children were asked to chew on a cotton swab) and explained the contents of the home pack (containing the 4-day food diary, Actigraph accelerometer and parent questionnaire). Children were instructed to return the home pack to school eight days after the measurement day for collection by a member of the study team. Class teachers distributed a reminder letter for parents two days before pack collection. Completion of packs was checked upon collection and follow-up of unreturned packs was conducted by the school administrative staff.

### Data collection and processing

The descriptions below relate specifically to the measures for which data are presented in this paper.

#### Aggregated school-level information

Norfolk County Council provided school-based summary data on all Local Authority (state) schools. This included school status (state vs. private), postcode, number of Year 5 pupils, proportion of all children receiving free school meals, ethnicity distribution and participation in the Healthy Norfolk Schools Programme [26]. Also provided was data from the 2007 Norfolk Primary Care Trust Child Height and Weight Survey, containing information on anthropometry for over 85% of Norfolk Year 6 children (aged 10–11 years). Data on independent private schools was provided by the Independent Schools Council or directly obtained from schools. Urban/rural classification for the schools was determined using Bibby and Shepherd's (2004) classification of rurality [27] and four density profiles, ranging from 'hamlet & isolated dwelling' to 'urban, more than 10,000 inhabitants', were used in the present study.

#### Demographic information

Children self-reported their date of birth, and age was calculated using the measurement date. Ethnicity was reported separately by both parents using the UK standard classification. Results were combined to determine the child's ethnicity, which was collapsed into a dichotomous variable (white vs. other ethnic background). The parent or main carer self-reported their highest educational qualifications (in categories) and home postcode to determine urban/rural location.

#### Anthropometry

Simple non-invasive anthropometry measures were conducted using standardized procedures with children dressed in light clothing. Portable Leicester height measures were used to measure height to the nearest millimetre. Waist circumference was measured twice to the nearest millimetre at the midpoint between the lower costal margin and the level of the anterior superior iliac crests, using a Seca 200 measuring tape. A third measurement was taken if a discrepancy of three or more centimetres was observed and an average was calculated. A 0.5 cm correction was applied to account for clothing [28]. A non-segmental bio-impedance scale (Tanita, type TBF-300A) was used to measure weight (to the nearest 0.1 kilogram) and impedance. Previously validated and published equations were used to calculate body fat percentage (BF%), fat mass (FM) and fat free mass (FFM) from the impedance value [29]. Body mass index (BMI) was calculated as weight (in kilograms) divided by height squared (in meters). Obesity status was determined using gender- and age-dependent cut points [30]. All scales were calibrated before and half-way through data collection. Quality assurance was established by assessing inter-observer variability of height and waist circumference before and after data collection, which was found to be acceptable (ranging from 0.1–0.3 centimetres for height and 0.7–1.3 centimetres for waist circumference).

#### Physical activity

Free-living physical activity was assessed over one week with the ActiGraph activity monitor (GT1M, Actigraph LCC, Pensacola, US). The children wore the accelerometer on an elastic waistband on the right hip during waking hours, except whilst bathing and during other aquatic activities. Children kept an Actigraph diary to report when they had taken the monitor off and for what reason. Activity data were stored at 5-second intervals and were downloaded to a computer upon receipt. In case of monitor failure or lack of data, children were asked to re-wear the monitor for a further week. All monitors were calibrated using a mechanical spinner before the start of data collection. A bespoke programme (MAHUffe, ) was used for data reduction and further analyses. The first day of data collection (day of measurement at school) was removed from all files and 10 minutes of continuous zero counts were classified as 'non worn time'. The outcome variables were daily activity counts per minute (cpm) and time (min) spent in moderate to vigorous intensity physical activity (MVPA, >2000 cpm). This threshold corresponds to a walking pace of about 4 km/h in children [31], and has been applied successfully in this age group before to study associations between physical activity intensity and metabolic outcomes [32]. Daily cpm is an indicator of the total volume of physical activity (i.e. average intensity of physical activity). This variable was derived by dividing total counts by monitoring time per day (between 6.00 and 23.00) and averaged over the measurement period. We have previously shown that this variable is significantly correlated with physical activity energy expenditure obtained by the doubly-labelled water method [33]. Children who did not manage to record valid data for at least 500 minutes per day on at least 3 days were excluded from further analyses.

#### Dietary behaviour

Food choice was assessed using an adapted version of the Health Behaviour in School Children (HBSC) questionnaire, a 15-item questionnaire of the most commonly consumed foods that has been previously validated in a Belgian sample and can be used for ranking participants for most food items [34]. Adaptations to the items included removing alcohol consumption, adding fruit juice consumption and separating sweets and chocolate into two separate items. Children reported on the frequency of consumption on a 7-point scale. Results were dichotomized at less than once per day ("never", "less than once per week", "once per week", "2–4 days per week", "5–6 days per week") vs. once or more per day ("once a day, every day", "every day, more than once") [35].

### Statistical analyses

Descriptive data were summarized as means with standard deviations or percentages, and differences between groups were tested using independent samples t-tests or Pearson Chi-square tests. Differences between activity levels on week- and weekend days were tested using paired t-tests, and Chi-square tests were used to test for differences between groups meeting the physical activity guideline or consuming at least one portion of fruit and vegetable per day.

## Results

### Recruitment

**Figure 1:**
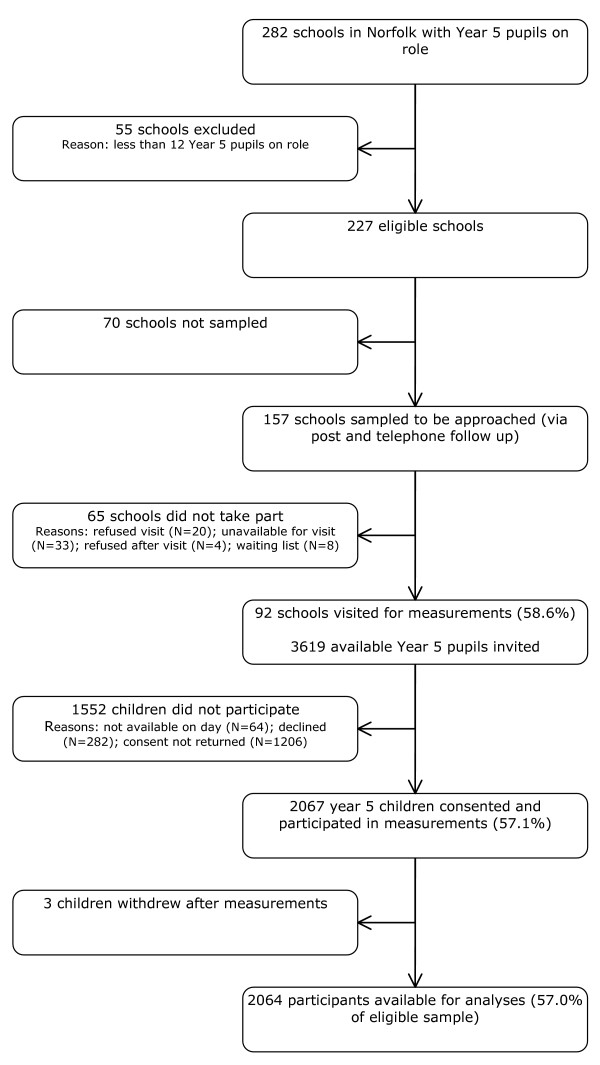
Flowchart of recruitment of schools and children into the SPEEDY study.

Figure 1 provides an overview of recruitment of schools and children. A total of 227 (214 state and 13 independent) schools were eligible for recruitment, of which 157 were sent an invitation letter. 101 Schools agreed to participate in the SPEEDY study and measurements sessions were conducted at 92 schools (58.6% of invited schools). At these 92 schools, 3619 Year 5 children were invited to take part (range per school: 5–155), measurements were performed on a total of 2064 children (57.0% of eligible sample) with response rates per school ranging from 13% to 100%. Of these, a total of 2043 children returned the Actigraph accelerometer (99.0%), with 1868 (90.5%) providing valid data. No differences were observed between children with and without valid physical activity data for BMI, overweight status, or parental education. However, girls were more likely to not provide valid data.

### Sample description

Table 1 provides an overview of the characteristics of participating schools. Compared with all eligible Norfolk schools, village schools were over-sampled in the SPEEDY study, with an under-sampling of schools in urban areas and of independent schools. Table 2 provides a description of characteristics of the SPEEDY children. A higher proportion of girls than boys participated and only a small proportion of children were from a non-white ethnic background.

**Table 1:** Characteristics of the schools participating in SPEEDY study compared with available data on eligible Norfolk schools.

	SPEEDY schools	All eligible Norfolk schools
School location		
- Urban (>10,000 inhabitants)	32.6%	34.7%
- Town and fringe	39.1%	38.0%
- Village	23.9%	20.7%
- Hamlet & isolated dwelling	4.3%	6.6%

Independent school	3.3%	7.5%

Free school meals (% children) (median)	9.0%	9.0%

Norfolk Healthy Schools participation (%)	38.2%	42.6%

Ethnicity (% non-British White) (median)	5.2%	4.6%

Weight status (%)*		
- overweight	13.4%	13.8%
- obese	16.7%	15.8%

No of year 5 pupils (median)	31	31

Sex (% boys in Year 6)*	52.5%	51.0%

NOTE: Figures based on data aggregated at school level. Data on Healthy Norfolk Participation, ethnicity, BMI and sex were only available for state schools, %Free school meals not applicable for independent schools.

*: assessed in Summer 2007 in Year 6 children (Norfolk Primary Care Trust child Height and Weight Survey).

**Table 2:** Descriptive personal and anthropometry data on the SPEEDY study sample (all numbers in cells are mean (standard deviation) unless stated otherwise)

	**Boys**	**Girls**	**Total**
N (%)	926 (44.9)	1138 (55.1)	2064

Age (years)	10.2 (0.3)	10.3 (0.3)	10.3 (0.3)

Ethnicity (% non-white)	3.8	3.8	3.8

SES – parental education (%)			
- GCSE or lower	36.3	40.6	38.7
- up to A level	42.2	40.4	41.2
- higher education	21.4	18.9	20.0

Home location			
- Urban (>10,000 inhabitants)	40.3	38.9	39.6
- Town and fringe	26.6	29.9	28.4
- Village	25.4	24.7	25.0
- Hamlet & isolated dwelling	7.7	6.5	7.0

Height (cm)	140.9 (6.5)	141.0 (6.8)	141.0 (6.7)

Weight (kg)	35.7 (7.7)	37.1 (8.8)*	36.5 (8.4)

Bodyfat%	27.2 (7.5)	33.2 (7.3)*	30.5 (7.9)

Waist circumference (cm)	63.6 (7.8)	63.6 (9.0)	63.6 (8.4)

BMI (kg/m2)	17.9 (2.9)	18.5 (3.4)*	18.2 (3.2)

Weight status (%)			
- overweight	15.0	19.3*	17.4
- obese	4.1	6.6	5.5

*Statistical significant difference between boys and girls at p ≤ 0.001

### Anthropometry

Table 2 describes the anthropometric data collected in the SPEEDY study. Compared with boys, girls were heavier, had less fat free mass and more fat mass, a higher body fat percentage and a higher BMI (all p < 0.001). In addition, a higher proportion of girls than boys were classified as either overweight or obese.

### Physical activity

**Figure 2:**
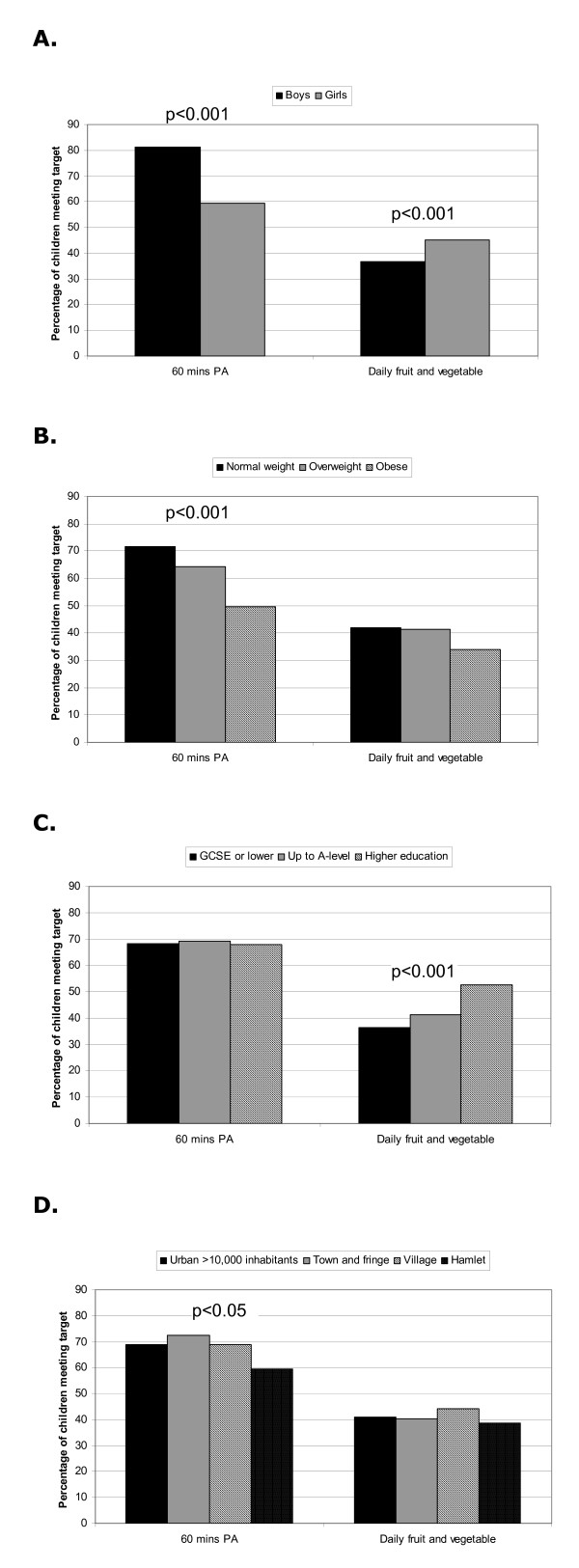
Percentage of SPEEDY children meeting physical activity (PA) guideline of 60 minutes per day on an average day of the week and reporting consuming at least one piece of fruit and one serving of vegetable per day, stratified by demographic characteristics (A. sex; B. weight status; C. parental education (indicator of socioeconomic status); and D. home location).

Table 3 describes the data on objectively measured physical activity stratified by gender. Overall physical activity and time (min/day) spent in MVPA was significantly higher (p < 0.001) in boys than girls. Both boys and girls did more activity overall on weekend days compared with weekdays. However, time spent at moderate and vigorous intensity activity did not differ between weekend and weekdays in either gender. Figure 2 shows that a higher proportion of boys than girls met the physical activity guideline, and that the proportion differs by weight status and home location

**Table 3:** Description of physical activity variables as collected with Actigraph accelerometers in the SPEEDY study.

	**Boys (N = 822)**	**Girls (N = 1046)**	**Total (N = 1868)**
Counts per minute			
- Weekday	682.6 (176.9)	592.9 (163.5)*	632.4 (175.3)
- Weekend day	754.8 (346.9)^#^	690.7 (343.9)*,^#^	719.6 (346.7)^#^
- Total week	716.5 (220.2)	635.6 (210.6)*	671.2 (218.6)

Minutes of MVPA			
- Weekday	83.5 (23.4)	65.5 (19.5)*	73.5 (23.1)
- Weekend day	85.1 (38.5)	67.2 (30.2)*	75.3 (35.3)
- Total week	84.1 (25.9)	66.1 (20.8)*	74.1 (24.9)

Differences between boys and girls tested with independent t-tests, differences between week and weekend day activity with paired samples t-tests.

*: difference between boys and girls at P < 0.001.

^#^: difference between weekday and weekend day activity within group at p < 0.001

### Dietary behaviour

Table 4 shows the results of the food choice questionnaire. Daily consumption of fruit and of vegetables was 56.8% and 49.9% respectively, with girls reporting daily consumption more frequently than boys. 41.3% of children reported eating at least one piece of fruit and one serving of vegetables per day, with significant differences between sexes (see Figure 2). This percentage also increased with increasing socioeconomic status, but did not differ by weight status or home location. It was also not associated with meeting physical activity guidelines (40.7% vs. 42.2% for whether or not participants were meeting the physical activity guideline, respectively). More than half of children reported daily consumption of skimmed or semi-skimmed milk, breakfast cereals and fruit juices.

**Table 4:** Dietary habits of the SPEEDY sample, as measured with food frequency questionnaire (numbers in cells are percentage of children reporting to consume the food type at least once a day)

	**Boys**	**Girls**	**Total**
Fruit	51.6	61.1*	56.8

Vegetables	44.8	54.1*	49.9

Sweets	18.4	12.1*	14.9

Chocolate	18.6	14.2*	16.2

Sugary soft drinks (e.g. coke)	16.1	9.3*	12.4

Diet soft drinks	16.2	11.7*	13.8

Skimmed/semi-skimmed milk	54.1	51.0	52.4

Whole fat milk	15.6	13.2	14.3

Cheese	17.4	16.4	16.9

Breakfast cereals	60.1	51.9*	55.6

White bread	44.0	41.7	42.7

Brown bread	22.3	20.0	21.0

Crisps	24.1	20.5	22.1

Chips	7.8	5.3*	6.4

Fruit juices	54.5	54.9	54.7

Differences between boys and girls tested with Pearson Chi-square.

*: differences between boys and girls at p < 0.05

## Discussion

The aim of the current paper was to present data on physical activity and dietary behaviour from a population-based sample of British 9–10 year old children. Results showed that more than two-thirds of children adhere to the physical activity guideline of accumulating at least 60 minutes of MVPA each day, but that daily consumption of fruit or of vegetables was only reported by 56.8% and 49.9% of the children. Boys were more likely to be physically active and of normal weight than girls. In contrast, boys' reported daily consumption of 'healthy' foods was lower, and their consumption of snacks and unhealthy food items such as soft drinks was higher than that of girls. In addition, normal weight children were more likely to meet physical activity guidelines than overweight and obese children, whereas children from a higher socioeconomic background were more likely to eat fruit and vegetable daily.

### Physical activity

Estimates of total physical activity, represented as average counts per minute, are largely comparable to the results of previous population-based studies in a similar age group in Britain and other European countries [15,16,36,37]. The small differences observed may be due to differences in monitors used and data processing strategies. However, it may also be due to the known age-related decline in physical activity [16,19], potential decreases in physical activity levels in the past decade [15,37], or seasonal effects [15,16,36]. As consistently reported before [15,16,19], boys were substantially more active than girls. In contrast to previous work, the data in this study were collected only during the spring and early summer. A seasonal effect on physical activity levels in children has previously been established [16,38,39], with lower activity levels observed during cold or extreme weather. Therefore, our estimate of overall physical activity may be overestimated compared with studies collecting data throughout the year. The timing of our measurements may also explain the observation that children were consistently more active on weekend days than on weekdays, in contrast to previous work [16,40]. During European spring and summer, children will be more likely to play outside for longer periods of time in their free time as a result of comfortable temperatures and longer hours of daylight. Time spent outside has previously been associated with higher activity levels [19].

In contrast to total activity levels, estimates of time spent in at least moderate-to-vigorous physical activity and of proportions of children meeting guidelines differ from other studies. Previous estimates of minutes of MVPA per day in this age group range from 20 to 192 minutes per day, with 2.5 to 97% achieving the recommended guideline of at least 60 minutes of MVPA each day of the week [13,15-18]. On average, the children in the SPEEDY study accumulated 74.1 minutes of MVPA per day, with 69.1% of children being classified as sufficiently active according to current physical activity recommendations. This value is higher compared with previous estimates in British children recently reported [16]. However, data on time spent at MVPA and prevalence of children being sufficiently active according to recommendations are not directly comparable between studies. This is explained by different thresholds used when defining MVPA, for which currently no consensus exists. Previously published thresholds for MVPA vary substantially depending on the activities performed during the calibration process, and the choice of intensity threshold will affect the results for MVPA substantially. This at least in part explains the discrepancy with previously published British data [16], where the authors used a threshold of 3600 cpm, a substantially higher threshold than used in previous work [15,17] and in the current study. This threshold (2000 cpm), however, lies mid-way between the various intensity thresholds derived in children, which range from 615 [41] to 3600 counts per minute [16].

One interpretation of our data could be that only one third of children at this age are insufficiently active. However, this interpretation may be dangerous as recent data suggest that about 90 to 120 minutes of moderate intensity physical activity is needed to prevent clustering of cardiovascular risk factors in children [6]. Given the well-established age-related decline in physical activity which appears to start before puberty [20,21], examining the correlates and determinants of physical activity and of changes in physical activity is therefore of particular interest in this age group in order to inform preventive action. Such interventions may be better placed if they focus on preventing declines in physical activity, and not necessarily increases in physical activity.

### Diet

A pattern of unhealthy food choices was revealed with high consumption of energy and fat dense food and refined carbohydrates. Our results however compare favourably to those from a previously reported HBSC survey in a slightly older population (11–15 year olds) in 35 countries. Here daily fruit and vegetable consumption ranged between 20–50% and 10–50%, respectively [24]. Results from 6081 participating English adolescents indicated that only 25% reported daily fruit consumption, with daily vegetable consumption just under 30%. In addition, about 40% of adolescents reported daily consumption of soft drinks, compared with 12% in the SPEEDY population. Whether these differences are due to age-related decline in healthy dietary habits, changes over time (the HSBC data was collected in 2002), reporting bias, or differences in populations studied, remains uncertain.

Results of the current study are largely in agreement with results from the National Diet and Nutrition Survey (NDNS) in children aged 7 to 10 years, conducted in 1997 [25]. In this study, for example, consumption of semi- and skimmed milk and white bread was also higher than whole milk and brown or wholemeal bread. Moreover, boys were less likely to regularly consume vegetables, whereas girls consumed less breakfast cereals than boys. In contrast, the children in the SPEEDY study reported a higher consumption of both artificially sweetened and sugary soft drinks, which may reflect changes in children's food consumption over time.

It is known that children's recall of food intake is prone to reporting errors [42,43] and that children's interest and enthusiasm may wane during lengthy dietary assessment periods. Therefore the short HBSC questionnaire was used to assess intake of the most commonly consumed foods rather than exact intake of nutrients, in order to collate information on trends in dietary intake. Under-reporting of energy intake has previously been reported in both boys and girls [44,45], with under-reporters reporting consuming the same number of fruit and vegetable servings, but fewer servings from sweets and fats groups [44]. Under-reporting is also associated with weight status [46], possibly explaining the observed healthier dietary profile in girls compared to boys given the differences in weight status by gender. As with the physical activity measurement, it is likely that seasonal influences have played a role in the assessment of dietary intake [47]. In particular fruit and vegetable availability and choice of food and drink consumption in warmer weather might have increased the reported consumption frequency of fruit, vegetables and soft drinks.

### Recruitment and representativeness

One of the aims of the sampling strategy in the SPEEDY study was to achieve maximum environmental heterogeneity in order to address questions relating to environmental influences on behaviour. In line with this we recruited a larger proportion of schools in rural than in urban locations. This might have affected the prevalence of certain behaviours amongst our school sample, and hence their representativeness to the wider population. On the other hand, as the other characteristics of the sampled schools are comparable to those of all eligible schools, it appears that we recruited a broadly representative sample of schools into our study.

Of all invited children, 57.0% took part in the data collection. Response rates may have been affected by the amount of data collected, the speed of the data collection (leaving ample time for additional reminder efforts), the recruitment method (only contact with children through schools) and previous negative research experiences in schools. No data is available on those children not taking part, reducing the potential to establish the representitaveness of the study sample. It does appear that girls are over-represented in the study and, compared to the Norfolk population, that a smaller proportion of obese children have taken part. Data using school-based assessments of obesity levels in Year 6 children across Norfolk in the same year indicate that 14.1% are classified as overweight and 15.8% obese [48]. It is well-established that non-responders to health surveys are more likely to have an unhealthy lifestyle, and this may have resulted in more positive prevalence estimates. Our final sample is mainly white with only 3.8% of children from other ethnic backgrounds. Although this is representative of the Norfolk population, it also reduces the generalizability of our results to the wider British population.

### Strengths and limitations of the SPEEDY study

Strengths of the SPEEDY study include the population-based sampling strategy, the large sample size, the use of objective and valid measures of physical activity and anthromopometry and of a frequently used and validated food frequency questionnaire. An intensive measurement period meant that measurements were only conducted during the Summer term. The advantage of this approach is the reduced influence of weather on the physical activity measurements and its potential confounding effect on the association with potential correlates. However, it may have affected the absolute levels of physical activity and diet intake presented. Other limitations include the use of a self-report measure of dietary intake, a suspected higher non-response of obese children and the fact that the demographic profile of the county of Norfolk is not representative for the whole of Britain.

The second aim of the SPEEDY study is to examine individual and collective factors associated with physical activity levels and dietary behaviour in a large population-based sample of British 9–10 year old children. The study is the first European study in children setting out to collect objectively measured physical activity and detailed dietary information together with a wide range of potential correlates from the psychological, biological, socio-cultural and environmental domains [49]. Future studies from the SPEEDY study will address these associations and provide much-needed information on where and how to target intervention efforts most effectively [50] in order to improve physical activity and dietary behaviours in children.

## Conclusion

The aim of the current paper was to provide descriptive data on physical activity and dietary behaviour of the children participating in the SPEEDY study. The results show that physical activity levels are relatively high, especially in boys, indicating that a prevention of decline, rather than increasing physical activity levels, might be a modifiable focus for intervention development in this age group. Although the dietary habits of the SPEEDY children seems to be better than reported in previous studies, a significant proportion of the children reported consuming limited intakes of fruit and vegetables. Therefore, promotion of daily fruit and vegetable intake in this age group is warranted, possibly focussing on children from lower socioeconomic backgrounds.

## Competing interests

The authors declare that they have no competing interests.

## Authors' contributions

EvS, PS, AJ, AC and SG were responsible for conception of the study, study design, set up and data collection and processing. KM coordinated the data collection. AMC, FH and JP participated in the data collection and processing. IH, UE and NW were involved with conception of the study and study design. EvS analyzed the data and drafted the manuscript. All authors were involved with data interpretation, critical revisions of the paper and provided approval for its publication.

## Pre-publication history

The pre-publication history for this paper can be accessed here:


